# Evaluation of different methods of environmental enrichment to control anxiety in dogs undergoing hemilaminectomy after acute intervertebral disc extrusion: a randomized double-blinded study

**DOI:** 10.3389/fvets.2023.1124982

**Published:** 2023-05-30

**Authors:** Ellery Pennington, Cary Springer, Julia Albright, Aude Castel

**Affiliations:** ^1^Small Animal Clinical Sciences, The University of Tennessee, Knoxville, TN, United States; ^2^College of Veterinary Medicine, The University of Tennessee, Knoxville, TN, United States

**Keywords:** post-operative analgesia, canine, essential oils, dog-appeasing-pheromones, music therapy, pain management

## Abstract

**Objective:**

The goal of this randomized, double-blinded, placebo-controlled study was to evaluate the effect of environmental enrichment (EE) on post-operative pain and anxiety in dogs following hemilaminectomy for acute intervertebral disc extrusion (IVDE).

**Methods:**

Twenty healthy client-owned dogs undergoing a hemilaminectomy for IVDE with the same immediate post-operative analgesia protocol were randomly assigned to either the EE or standard environment (SE) group post-operatively. Recovery was achieved in an intensive care room (SE) or a separate quiet room (EE) equipped with white noise and classical music. EE dogs were also exposed to dog-appeasing pheromones, essential oil scents, and positive human interactions and were provided meals through food toys. A blinded evaluator assessed all dogs using the modified Glasgow Composite Pain Scale (mGCPS) on presentation and at several time points post-operatively. A rescue injection of the opioid methadone was given to the dogs with an mGCPS score of ≥5/20. Dogs received the antidepressant trazodone when anxious behaviors (5 mg/kg) were observed. The mGCPS scores, the latencies to receive the first methadone and trazodone doses and to eat the first meal, the number of methadone and trazodone doses, and the number of meals ingested in the first 24 and 48 h post-surgery were compared using Wilcoxon tests, and Benjamini–Hochberg correction for false discovery rate was applied.

**Results:**

Although median mGCPS scores did not differ between the groups, compared to SE dogs (*n* = 10), EE dogs (*n* = 6) received trazodone earlier (*p* = 0.019), were administered fewer methadone injections at 24 h (*p* = 0.043), and ate more at 48 h post-surgery (*p* = 0.007). Therefore, EE and anti-anxiety medications could be beneficial in improving the wellbeing of dogs post-operatively.

## Introduction

Multimodal analgesia, which refers to the application of a combination of multiple analgesic drugs and/or techniques with different targets along the pain pathway, should be adopted as the standard of care in veterinary medicine. Indeed, the rationale for using perioperative multimodal analgesia is that it not only optimizes the use of analgesia using additive or synergetic effects produced from different analgesics but also allows for dose reduction, thus minimizing potential side effects, such as sedation, decreased appetite, vomiting, dysphoria, and constipation (1–4). Therefore, appropriate perioperative pain management strategies should rely on multimodal analgesia protocols that act on both peripheral and central targets (5). Although a combination of two or more different classes of analgesics can be used for this purpose, in recent years, studies looking at non-pharmaceutical approaches to modulate pain have flourished, and animal models are commonly used to evaluate their efficacy (6–8). Environmental enrichment (EE) is one of these methods and can be applied in the form of social, cognitive, physical, sensory, or even nutritional enrichment. For example, studies on rodents using a chronic neuropathic pain model found that EE partially alleviated pain (9). EE and social enrichment were also found to decrease the use of self-administered analgesia following cecal manipulation in mice (10). Exposure to classical music, another enrichment method, was found to enhance the antinociceptive activity of analgesic drugs in an inflammatory and surgical pain model in this species (11). In contrast, in one study, EE decreased anxiety in adult animals but had no effect on pain (12). In humans, EE in the form of music therapy was shown to reduce post-operative pain and anxiety associated with surgical intervention (13, 14). Aromatherapy was also found to be beneficial in reducing post-operative pain and anxiety (15, 16). Companion animals were also found to be influenced by EE, including human contact, music, odors, or toys, and if these modalities positively modulated their anxiety, they could also be potentially beneficial for pain as both conditions (referring anxiety and pain) share the same neurophysiological mechanisms and can influence each other (17–21). To our knowledge, to date, no study has looked at the effect of EE on post-operative anxiety and pain in companion animals and more specifically, in dogs. The present study aimed to evaluate the effect of different methods of EE (social, sensory, cognitive, and nutritional) on post-operative pain and anxiety in dogs following hemilaminectomy for acute intervertebral disc extrusion (IVDE). We hypothesized that an enriched and low-stress environment would reduce the need for rescue opioid administration and decrease anxiety, thus helping to improve the return of appetite in dogs that have undergone spinal surgery for an intervertebral disc herniation causing neurologic deficits.

## Materials and methods

The present randomized, double-blinded, placebo-controlled study was conducted on 20 client-owned dogs (8 in the EE group and 12 in the SE group) presented to the Neurology Service of the University of Tennessee, College of Veterinary Medicine for neurological deficits suspected to be secondary to an acute intervertebral disc extrusion (IVDE). The owner's consent to have their dog participate in the study was acquired at the time of admission. Dogs were assessed using a modified Glasgow Composite Pain Scale (mGCPS) prior to surgery and then randomly assigned to either the environmental enrichment (EE) or standard environment (SE) group post-operatively. The method of randomization relied on the use of a simple random sampling formula in Excel to determine the order of the cases with the random drawing of an envelope, in which the name of the assigned groups was written on pieces of paper and kept in sealed envelopes (numbered from 1 to 20), in chronological order, at the time of recruitment. The drawn envelope was presented to the clinician overlooking the study of the post-operative environment to inform them of the group to which the patient was assigned.

This protocol was approved by the Institutional Animal Care and Use Committee of the University of Tennessee (protocol number 2604_0418).

### Inclusion and exclusion criteria

To fulfill the inclusion criteria, only the dogs that had had neither back surgery nor an episode of back pain or neurologic deficits previously were included. The clinical signs spanning a duration of < 2 weeks were expected to be acute for the dogs to be included in the study. These criteria were chosen to avoid any component of chronic pain that could affect the pain management plan. The dog's demeanor had to allow for regular pain assessments and interactions without aggression or excessive fear. Dogs with a body condition score >7 on a scale of up to 9 were excluded, as it was assumed that obesity could affect the surgical time (with more adipose tissue to dissect) and may contribute to increased pain, as shown in humans with a spinal disease (22). Brachycephalic dogs were also excluded due to the increased risk of complications associated with stenotic airways, requiring them to be more closely monitored in an intensive care unit (ICU) room. Dogs with comorbidities requiring additional treatments and/or monitoring were also excluded. Finally, dogs with neurological deficits not associated with IVDE and dogs in which IVDE was extensive, requiring a multiple-site hemilaminectomy (and therefore a longer and potentially more painful surgery), were excluded.

### Blinding of the study

The evaluator was completely blinded to the study protocol. The purpose of the study was not revealed to the evaluator, and she was informed that she was participating in a study that aimed to assess the effectiveness of different methods of measuring pain and a different analgesic protocol, but she did not know precisely what was being looked at. Since, generally, the dogs were allowed to recover either in the wards (quiet room) or the ICU following a hemilaminectomy, it was difficult to determine which dogs were part of the EE study and which dogs were not when the enrichment methods were removed (just before the assessments).

The clinician in charge of the study was unblinded and was responsible for providing the enrichment conditions in the room and removing them before each assessment by the blinded evaluator.

The clinician in charge of the case made the decision on whether or not the dog needed to be excluded because of pain based on the mGCPS score measured by the evaluator or the response to treatment (rescue methadone and for some, trazodone).

### Perioperative care

Dogs were anesthetized to undergo computed tomography (CT) or magnetic resonance imaging (MRI) to confirm the presence and location of an IVDE using a standardized anesthesia protocol (Table 1). Dogs were kept under anesthesia and taken to surgery immediately after imaging. All dogs had a routine hemilaminectomy performed under a standardized analgesia protocol (Table 1). The surgery was performed by different experienced surgeons and neurosurgeons, depending on who was on clinic duty and potentially on call at that time.

**Table 1:** Standard anesthesia and analgesia protocol followed for all patients included in the study.

	**Drug**	**Route of administration**	**Dose range**
Premedication	Midazolam	IM or IV	0.3–0.5 mg/kg
	Fentanyl	IV	5 ug/kg
	+/-Dexmedetomidine	IV or IM	3–5 ug/kg
	Maropitant	IV or SQ	1 mg/kg
Anesthesia	Propofol	IV	1–4 mg/kg
Surgery	Isoflurane	Inhalant	
	Fentanyl	IV (CRI)	3–10 ug/kg/hr
Recovery	Methadone	IV	0.2 mg/kg
	Dexmedetomidine	IV	1 ug/kg
	Carprofen	SQ	2.2 mg/kg
Post-operative analgesia	Gabapentin	PO	10 mg/kg q 8h
	Carprofen	PO	2.2 mg/kg q12h
	Diazepam	PO	0.5 mg/kg q8h

CRI, continuous rate infusion; IM, intramuscularly; IV, intravenously; PO, per os; SQ, subcutaneously. The fentanyl was tapered off approximately 20 min after the dose of methadone was given following extubation. Dexmedetomidine was given at extubation when dysphoria was noted. A non-steroidal anti-inflammatory injection was given if the dog did not encounter significant hypotension during the procedure or had received prior corticosteroid administration.

### Post-operative care

All dogs received the same immediate post-operative analgesia protocol (Table 1). One dose of methadone was administered intravenously to all the dogs at the time of extubation, and then, all dogs were started on oral pain medications immediately after they were awake enough to swallow the medicine safely, which was approximately 4 h following extubation. Dogs were placed in a size-appropriate metal cage with soft bedding for recovery within the ICU room (SE group) or a separate quiet room in another area of the hospital (EE group). The EE room was also equipped with a white noise machine to mask sounds originating from outside the room, low-volume and low-tempo classical music (Through a Dog's Ear? and Classical music for dogs?) was played for at least 8 h per day, and dog-appeasing pheromones (Adaptil?) were applied to the bedding three times daily. A few drops of essential oil (lavender on day 1 and chamomile on day 2) were applied on a gauze placed in a little container in front of the cage door. Finally, the dogs were provided with human affection sessions (5 min) three times daily, with the caretaker offering treats and petting and talking to them. All dogs were evaluated by technicians or 4th-year students in a veterinary school every 4 h to monitor their heart rate, respiration rate, temperature, and overall attitude. Dogs that were paralyzed and could not urinate on their own had their bladder expressed manually every 8 h. The surgical incision was iced every 6 h for approximately 5 min. Dogs demonstrating agitated behaviors, such as whimpering, vocalizing, and excessive panting, and appearing fearful but showing a pain score of < 5/20 or continued restlessness despite the administration of analgesics received a rescue dose of trazodone (5 mg/kg), which could be administered every 8–12 h if the anxious behavior reappeared. The dogs were taken for a walk outside (with a sling under the belly if non-ambulatory) every 6 h, and on both pelvic limbs passive range of motions was performed for 5 min every 8 h from 12 h after surgery.

The dogs of the SE group were fed Hills I/D or Purina E/N every 8 h in a paper bowl, while the dogs of the EE group received a similar diet in a food toy. If the dog was not interested in eating with the food toy, it was offered food in a regular bowl. The amount of food offered to each dog at each meal [one-third of daily resting energy requirement (RER)] was weighed initially before offering to the dog and after the meal was completed, and the eaten amount was recorded. Dogs in the EE group were also offered small amounts of various treats, such as peanut butter, canned cheese, or beef-flavored dog treats, during the human affection sessions.

### Pain and anxiety assessments

All dogs were evaluated by an evaluator blinded to the whole purpose of the study and the dog's environment using the mGCPS at the time of presentation and at several time points after surgery (4, 8, 12, 24, 36, and 48 h) before receiving any injectable drug. We used only five of the six categories included in the mGCPS, as the mobility section could not be reliably assessed because some of the dogs involved in the study were paralyzed (Supplementary material). The mGCPS assessment generated a score out of 20, and dogs with a score equal to or higher than 5/20 were considered to be in pain and were given additional analgesia in the form of a rescue injection of an opioid (methadone 0.2 mg/kg) in addition to the oral pain medications given as part of the standard protocol. Additional pain assessments were performed at the clinician's discretion if there was any concern that the dog could experience pain between the standard assessment time points. The dog was removed from the study if it was still reactive to touching the incision or looking at the wound and/or appeared hunched or tensed when moving around [criteria C and D (vi) of the mGCPS] even after being given the rescue dose of methadone. However, if the dog was still crying or whimpering and/or nervous or fearful [criteria A (i) and D (v)], an oral dose of trazodone (5 mg/kg) was given. If the aforementioned signs persisted despite this intervention, the dog was excluded from the study and received either more intravenous analgesia as a continuous infusion or anti-anxiety medications depending on the clinician's perception of whether the dog was in pain or anxious.

### Data collection and outcome assessment

To evaluate the primary outcome, i.e., whether EE decreased the need for opioids during the 48 h of hospitalization, the following data were collected: the mGCPS scores at 4, 8, 12, 24, 36, and 48 h post-surgery, time to the first injection of methadone (in hours), and the total number of methadone doses in 24 h and 48 h. Information regarding any additional pain and anxiety assessments performed was also collected. If a dog was excluded because of pain before the end of the 48-h evaluation period, the number of doses of methadone received was corrected by adding the number of doses that the dog would have received if it was determined as being in pain at each remaining pain assessment time point.

For the second outcome, i.e., whether EE decreased anxiety and, therefore, the need for anti-anxiety medication, the latency to receive trazodone (in hours) and the number of doses of trazodone received in 24 and 48 h following surgery were recorded.

For our third outcome, i.e., whether EE improved the return of appetite, the latency to return of appetite (in hours) (i.e., the time taken by the dog to start eating after surgery), and the number of meals eaten in 24 and 48 h following surgery were recorded.

### Statistical analysis

Statistical analysis was performed with the JMP Pro 17.0.0 software (SAS Institute Inc., Cary, NC). The sample size was calculated using the data collected for the first six cases (three dogs per group) as part of a pilot study. In accordance with the one-way analysis (two groups) of the total number of methadone doses received during the 48 h post-surgery, the following assumptions were made: α = 0.05, a power of 90%, an estimated standard deviation of 0.65, and a difference in mean characterized by a variance of means of 2. These assumptions indicated a sample size of 10 in each of the two groups (20 cases in total). We, therefore, decided to recruit cases until this number was reached.

The descriptive data of dogs in each group were compared using the Mann–Whitney *U*-test for continuous data and by constructing contingency tables and performing a chi-squared test for categorical data (breed and sex). A contingency table was used to determine the probability of being excluded for pain depending on the environment. The contingency table was then tested for independence by performing a Pearson's chi-squared test.

Wilcoxon tests were used to evaluate the differences in non-parametric variables between the groups. The following variables were assessed across the two environments: age, weight, BCS, median mGCPS scores at baseline, 24 h, and 48 h following surgery, need for rescue opioids (time to first injection and the total number of doses in 24 and 48 h) anti-anxiety drug (time to first administration and the total number of doses in 24 and 48 h), and return of appetite (time to first meal and the total number of meals in 24 and 48 h), across the two environments. A *p*-value equal to or < 0.05 was considered statistically significant. To correct for multiple comparisons, the Benjamini–Hochberg (B-H) correction was applied using a false discovery rate of 20%. The critical value was calculated for each individual *p*-value, allowing us to determine the significant variables.

## Results

A total of 20 3- to 9-year-old dogs with neurological deficits following thoracolumbar IVDE (confirmed by advanced imaging) and requiring surgery were recruited in the present study. However, four of them were excluded from our data analysis. One dog was excluded because the standard analgesia protocol was not followed, as gel foam soaked with morphine was used during surgery to cover the spinal cord prior to closure, which could have affected the pain level. This dog was excluded from the EE group. Three dogs (one in EE and two in SE groups) were excluded because of a lack of details regarding pain and anxiety assessments performed between the time points and potential biases associated with drug administration.

Therefore, a total of 16 dogs were included in our data analysis.

One dog in the SE group was excluded because it remained anxious despite trazodone administration and had to be given acepromazine. Three dogs in the SE group were excluded because of pain; however, they were still included in our data analysis, but the number of methadone doses was corrected as previously explained. When comparing the two groups for the probability of being excluded for pain or anxiety depending on their environment, the difference was found to be significant (*p* = 0.033).

The breed, age, sex, weight, and body condition score (BCS) of the participating dogs are summarized in Table 2 and did not differ significantly between the groups.

**Table 2:** Comparison of the signalment of dogs included in the environmental enrichment group and standard environment group.

	**Environmental enrichment (*n =* 6)**	**Standard environment (*n =* 10)**	***P*-value**
Breed	6 Dachshunds	6 Dachshunds	0.07
		1 Beagle	
		1 Pekingese	
		2 Mix breed	
Median age (yr) (SD)	5 (4–7)	4, 5 (3–9)	1.00
Sex	5 FS; 1 MC	5 FS; 5 MC	0.18
Median Weight (kg) (SD)	6.9 (4.6–7.6)	6.95 (5–17.3)	0.59
Median BCS (SD)	5.5 (4–7)	5 (4–7)	0.57

BCS, body condition score; FS, female spayed; MC, male castrated; MI, male intact; SD, standard deviation. A *p*-value equal to or < 0.05 is considered significant. None of the factors evaluated differed between the two groups.

The results obtained for the other variables are presented in Tables 3, 4.

**Table 3:** Results of the data analysis comparing the environmental enrichment (EE) group with the standard environment (SE) group.

	**EE (*n* = 7) (median and range)**	**SE (*n* = 10) (median and range)**	***P*-value**
Baseline mGCPS score	3 (1–6)	3 (1–7)	0.86
Median mGCPS score at 24 h	3.5 (2.5–5.5)	4 (2–5)	0.95
Median mGCPS score at 48 h	2.75 (2–4)	4 (1–5)	0.51
Time to 1^st^ methadone dose (h)	2 (2–4)	4 (2–9)	0.39
Number of methadone doses in 24 h	2 (0–3)	3 (2–5)	0.043^*^
Number of methadone doses in 48 h	3 (0–3)	4 (2–7)	0.11
Time to 1^st^ dose of trazodone (h)	5 (3–16)	16 (8–30)	0.019^*^
Number of trazodone doses in 24 h	1 (1–3)	1 (0–1)	0.08
Numbers of trazodone doses in 48 h	1.5 (1–6)	1 (0–5)	0.20
Time to 1st meal (h)	5.25 (2–26)	11 (3–29)	0.43
Number of meals eaten in 24 h	2 (1–3)	1 (0–2)	0.07
Number of meals eaten in 48 h	6 (3–7)	1 (0–5)	0.007^*^

A *p*-value equal to or < 0.05 is considered significant (and marked^*^).

**Table 4:** Benjamini–Hochberg (B-H) correction for multiple comparisons with a false discovery rate of 20%.

	***p*-value**	**Rank**	**B-H critical value**
Number of meals eaten in 48 h	0.007^*^	1	0.029
Time to 1st dose of trazodone (h)	0.019^*^	2	0.057
Number of methadone doses in 24 h	0.043^*^	3	0.086
Number of meals eaten in 24 h	0.07	4	0.114
Numbers of trazodone doses in 24 h	0.08	5	0.143
Number of methadone doses in 48 h	0.11	6	0.171
Number of trazodone doses in 48 h	0.2	7	0.200
Time to 1^st^ methadone dose	0.39	8	0.229
Time to 1^st^ meal (h)	0.43	9	0.257
Median mGCPS score at 48 h	0.51	10	0.286
Baseline mGCPS score	0.86	11	0.314
Median mGCPS score at 24 h	0.95	12	0.343

The *p*-values were reorganized from the smallest to the highest value and given a rank. To calculate the critical value to establish whether the *p*-value was significant, the following formula [(rank/number of tests) × 0.2] was used. A *p*-value equal or less than the corresponding B-H critical value are considered significant (and marked ^*^).

### Pain assessments and exclusion for pain or anxiety

The median mGCPS scores at admission (baseline), 24 h, and 48 h were not statistically different between the two groups (*p* = 0.95 and *p* = 0.51, respectively). A total of 8 dogs (two in the EE group and six in the control group) had additional blinded pain assessments because they experienced pain outside the experimental time points, with the majority of them being performed within the first 24 h following surgery. Four dogs (all in the SE group) had one additional assessment, three dogs (two in the SE group and one in the EE group) had two additional assessments, and one dog (one in the SE group) had three additional assessments.

Three dogs in the SE group were excluded owing to their failure to respond to rescue methadone (i.e., the persistence of an mGCPS score equal to or over 5/20) at 4, 8, 11, and 15 h following surgery. No dog in the EE group was excluded for persistent pain after rescue methadone injection or for anxiety.

### Results of methadone administration

**Figure 1:**
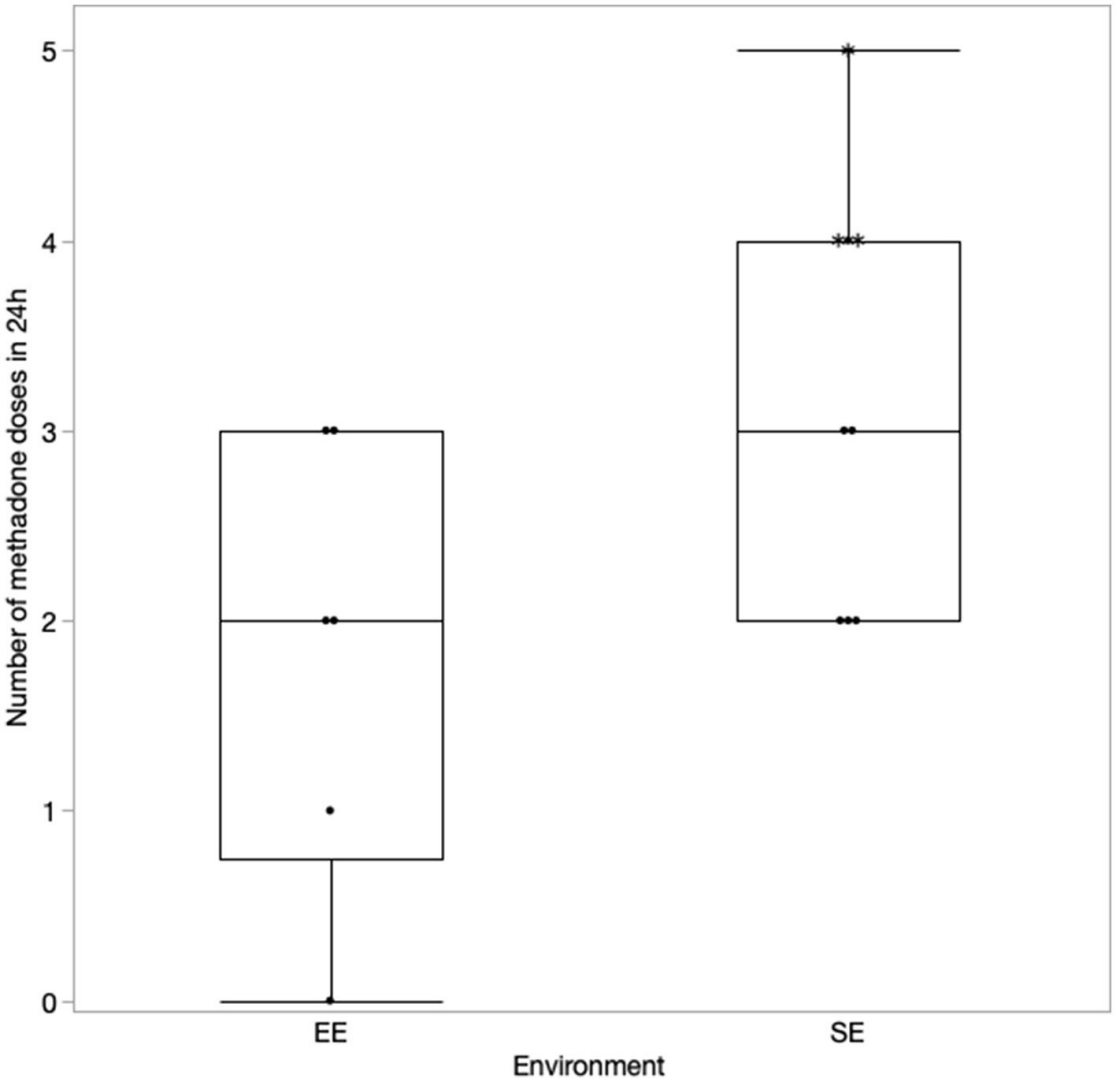
Number of methadone doses in 24 h with the correction made for the excluded cases (indicated by*).

**Figure 2:**
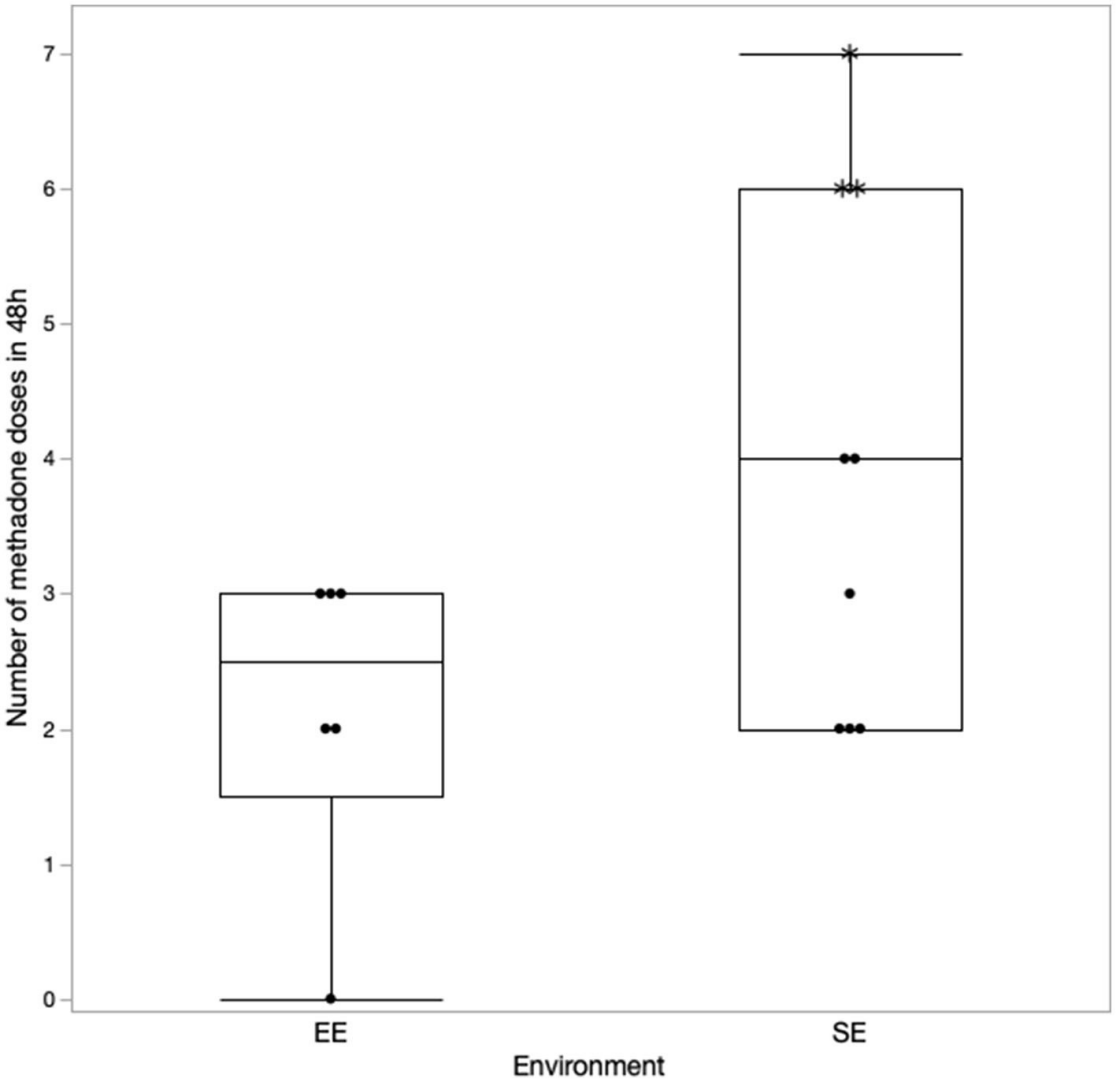
Number of methadone doses received in 48 h with the correction made for the excluded cases (indicated by*).

The details regarding the number of doses of methadone received by both the groups in 24 and 48 h are presented in Figures 1, 2, respectively. One dog in the EE group did not require any additional doses of methadone because it was found to be comfortable with only oral pain medications at each assessment time. The latency to receive the first injection of methadone was not different between the groups (*p* = 0.39). There was a difference in the number of doses received within the first 24 h (*p* = 0.043) (Figure 1) but not at 48 h (*p* = 0.11) post-operatively between the groups (Figure 2). The difference at 24 h remained significant when correcting for a false discovery rate of 20% (*p* = 0.043 < 0.086).

### Results of trazodone administration

One dog in the SE group had to be excluded at 5 h post-extubation because it was too anxious despite the administration of trazodone and was given an injection of acepromazine.

Dogs tended to receive trazodone much earlier than those in the SE group [EE: median latency 2 h (2–4); SE: 4 h (2–9), *p* = 0.019]. This difference was lower than the B-H calculated value and was, therefore, considered significant. However, there was no difference in the number of doses of trazodone received between both groups at 24 and 48 h post-surgery (*p* = 0.08 and *p* = 0.2, respectively).

### Results of meal intake

Two dogs in the SE group did not eat during the period of their hospitalization. One of them was excluded because of persistent pain despite the administration of a rescue methadone injection. There was no difference in the latency to eat the first meal between the two environments (*p* = 0.43). There was also no difference in the number of meals eaten within the first 24 h following surgery (*p* = 0.07). However, dogs in the EE group ate more meals than dogs in the SE group at 48 h following surgery (*p* = 0.007). After correction for the false discovery rate, this result remained significant, as the *p*-value was lower than the calculated B-H critical value of 0.028.

## Discussion

In this prospective placebo-controlled study, dogs recovering in an EE received fewer doses of rescue pain medication within the first 24 h post-surgery. Dogs recovering in an EE also ingested more food in the 48-h post-operative period compared to dogs recovering in an SE. These dogs also received the anti-anxiety drug trazodone earlier, but they did not need more doses during the overall assessment period. Although three dogs were excluded for pain and one for anxiety in the SE group and none in the EE group, the difference was not significant in terms of pain score and total doses of rescue methadone and trazodone. These findings raise multiple questions since pain is a complex experience that can be influenced by different factors, such as the patient's anxiety level and the person assessing it.

The median mGCPS scores did not differ significantly between the groups; however, in the SE group, three dogs were excluded because they remained in pain (i.e., reactive to touching their incision after methadone injection) and one dog was excluded for anxiety because it continued to vocalize despite the administration of trazodone. We attempted to minimize the bias as much as possible by having a blinded evaluator take the assessments and having precise criteria to determine whether a dog should be excluded or not. It is possible that averaging the scores over a certain period to compare the two populations might not be ideal because dogs could have overall similar scores, but at one time point, one dog could have a much higher score and respond poorly to the treatment, leading to its exclusion without being reflected in the median assessments. Exploring a continuum of pain level and anxiety level rather than isolated points would probably have provided a much better idea of whether these two groups were truly similar in terms of pain and anxiety. One population (potentially the SE dogs) might have experienced more pain, and the other population (EE dogs) might have been more anxious, but since the mGCPS scores can be affected by both conditions and because anxiety influences pain perception and pain can lead to anxiety, a difference might not be apparent (21). Indeed, dogs in the EE group did receive trazodone much earlier, which might suggest that they experienced anxiety-associated behaviors earlier than the SE dogs, and their mGCPS scores could have reflected this observation. The low number of cases included in the study might have also prevented the appearance of a statistical difference between the groups. Reliable methods of pain assessment, not affected by the dog's anxiety level, are needed to improve patient care and distinguish between pain and hospital fear.

This study found a beneficial effect of EE on dogs' appetite, as shown by a higher number of meals eaten post-operatively. Of note, we did not account for the additional unquantified amounts of food eaten in the form of treats as it was difficult to determine; thus, the results might have been even more significant. The small treats given by the caretaker as part of the positive interactions might have played a role in stimulating the dogs' appetite. It is also possible that, because these dogs received fewer rescue opioid doses in the first 24 h, they felt less nauseous and were, therefore, more willing to eat. However, we would probably have observed a difference in food intake at 24 h if this was the reason. It is also possible that earlier administration of trazodone favored better control of their anxiety and their overall wellbeing, which further influenced their food intake, as it is not uncommon for dogs, even when not submitted to painful procedures, to not eat in the hospital (23).

The effect of EE on analgesic use and appetite is probably multifactorial and likely affects both anxiety and pain by acting on the descending inhibitory pathways via the modulation of GABAergic mechanisms, whose disruption may cause cognitive deficits, anxiety, depression, and alteration in emotional modulation of pain (24).

The music that was played to the dogs as part of the EE protocol was a compilation of two classical music CDs specifically designed for dogs. Although this specific type of music was not found to be superior to random classical music played in previous studies (25, 26), it was chosen for consistency and study reproducibility. Other types of auditory stimulation should be studied, notably audiobooks, as this format of human speech was shown to decrease stress-associated behavior in kenneled dogs to a higher degree when compared to other auditory stimulation programs, including classical music (26). Furthermore, studies have shown that, as in the human ICU, the sound level in the veterinary ICU was relatively high, with the potential to disrupt patients' sleep, and that the use of white noise/sound-blocking devices improved the quality of sleep in the ICU patients (27, 28). With these facts in mind, patients in the EE group were kept in a quiet room equipped with a white noise machine to block the surrounding sounds and to improve rest and sleep. In retrospect, it would have been interesting to record and compare the time spent resting/sleeping between the two groups.

Aromatherapy with essential oils was used as part of the EE program because different studies had shown their beneficial effects on both anxiety and pain. Aromatherapy was also shown to decrease post-operative pain and analgesic demand in people, particularly following very painful procedures, such as sternotomy or cesarian section (16). Lavender oil, specifically, was shown to decrease pre-operative anxiety in people (15). In animals, the essential oil obtained from *Cedrus atlantica* was shown to have an anti-hyperalgesic effect in a mouse model of post-operative pain, and lavender oil was found to reduce neuropathic pain to an extent similar to pregabalin in rats (29, 30). In dogs, olfactory stimulation with essential oils, lavender and chamomile oils in particular, was found to improve stress-related behaviors in shelters (31, 32). If this type of olfactory enrichment seems beneficial to modulate anxiety in the canine patient, more studies are needed to evaluate the effect of essential oils on pain and to determine if some types of essential oils are more efficient than others. The type of oil was changed between the first (lavender) and second (chamomile) post-operative days to prevent habituation, which, in retrospect, might not have been necessary, as habituation was not observed even after 5 days in shelter dogs (32).

Dog-appeasing pheromones (DAPs) are pheromonal analogs of the appeasing pheromones that are secreted by nursing bitches. Dogs in the EE group were exposed to a commercial DAP sprayed on the bedding as it was shown to decrease the signs of hospital-induced anxiety (23), although results have been inconsistent among studies (33). To the authors' knowledge, to date, no study has determined their effect on the perioperative environment in dogs. Pheromones are perceived by the vomeronasal organ (VNO), which transmits information via the vomeronasal nerve to the amygdala, which belongs to the limbic system. Although the precise mechanism of action of pheromones is unknown, the rationale for using them as part of the behavioral modification program is that they could modulate the emotional status of an animal and inhibit some uncomfortable behaviors, such as fear reactions (34). However, for pheromones to be perceived, the VNO needs to be open, which is voluntary and usually influenced by the animal-specific perceiving behaviors performed by the emitting of individual messages that these pheromones are being expelled. Therefore, to ensure that a dog will perceive the appeasing pheromones, we need to expose the animal to higher amounts of pheromones than the required amount in the hope that it will open the VNO and be receptive to it (34). In a hospital, other types of smells (urine, feces, disinfectant, and anal gland secretions released by fearful animals) could potentially cover the smell of the DAP and hinder their beneficial effects. A lack of perceived efficacy of DAP in a study could be attributed to either the insufficient quantity of the compound (not sufficient for the animal to smell it) or the actual lack of effect.

Dogs in the EE group were also provided with positive interactions in addition to their regular treatment regimen. Dogs are highly social animals whose welfare is enhanced by human interactions (17, 35), but when interactions are limited to stressful and unpleasant procedures, such as manipulation for examination or treatment administration, it could create negative associations with people, exacerbate anxiety, and potentially increase the risk of fear aggression in dogs (20), which could be the reason why the dogs in the EE group received trazodone much earlier than the SE group. The dogs in the EE group might have been more anxious initially before realizing that these human interactions were meant to be positive. Additionally, the mGCPS requires that the animal be touched to assess its reaction to touching the surgical wound, and this type of manipulation could be distressing to some dogs. Therefore, in a dog with a low anxiety threshold associated with the anticipation of pain or unpleasant handling, pain assessments that do not rely on physical interactions with the dog could be more accurate. Although these periods of positive interactions could potentially increase distress through the occurrence of “white coat syndrome” or, conversely, by creating separation-related distress outside the affection sessions in some patients, we believe that positive interactions are more likely to decrease overall fear and stress in the hospital by showing the dog that not all interactions with their caretakers are unpleasant. This hypothesis is supported by a study showing that pleasant physical contact, such as grooming, lowered the heart rate, which can be viewed as a sign of physiological relaxation in dogs (35). Furthermore, dogs appeared calmer and had lower glucocorticoid levels when exposed to a novel environment in the presence of their caretaker (36). In retrospect, it would have been interesting to include a third group of dogs that would have been kept in an EE but without social interaction with humans to fully evaluate its effects on post-operative pain and anxiety in dogs (whether it increases or decreases anxiety). It would also be interesting to compare the effect of human interaction with familiar (owner) and unfamiliar individuals during hospitalization. It is possible that, in the context of a patient that requires some human intervention to administer treatments or procedures that can be unpleasant or even painful, adding human contact with unfamiliar persons, even if positive, could create more stress and should, therefore, be minimized.

Anorexia can be considered a sign of anxiety in a hospital environment (23), and toys, including some containing food, are recommended to decrease anxiety in dogs (17, 37). Therefore, food was offered in a toy as another form of enrichment. However, because not all dogs were willing to eat in this manner, food was also offered in a regular dog bowl. Unfortunately, we were not able to retrieve the information to determine how many dogs ate with the toy, the regular food bowl, or both.

This study has several limitations, particularly due to the small sample size. Unfortunately, we had to exclude some dogs either due to missing information concerning pain and anxiety assessment, which could have represented a bias in the data, or because the standard analgesia protocol was not respected. The loss of these cases could have affected the power of our study and our ability to see some differences between the groups.

Further larger-scale studies are therefore needed to confirm our results and to evaluate the effect of perioperative EE, anxiety treatment, and positive human interactions on dogs by following more routine surgeries performed by general practitioners, such as spaying, neutering, or dental procedures.

Since the surgical procedure could be performed by different surgeons, standardizing the surgeon performing the procedure would have been difficult. This limitation would have greatly affected our ability to recruit cases in a timely manner, as it would have been impacted by who was on clinic duty and potentially on call at that time because some of the cases were presented on an emergency basis. We attempted to minimize the variability between surgeons by ensuring that the same surgical approach/technique was performed in all the cases and taking care that the hemilaminectomy procedure was performed only by experienced surgeons.

Pain and anxiety were assessed using subjective criteria, which could have impacted the decision to administer methadone or trazodone. However, this bias was minimized by allowing a single person to assess all the dogs. This person was blinded to the general purpose of the study and did not know that keeping the animal in different rooms was part of the experiment. Additionally, the music-playing device, food toy, essential oil gauze, and white noise machine were all removed from the room before assessments. It cannot be ruled out that, even though the evaluator was blinded, she might have been influenced to some degree by the different environments and might have interacted differently with the dogs. Anecdotally, some personnel involved in the care of these patients reported feeling calmer in the EE room, and classical music has been reported to have a positive effect on employee satisfaction (25). This factor would be difficult to control, but it was minimized by removing most of the enrichment methods at the time of assessment. It would be interesting to evaluate the effect of EE using more objective measurements of anxiety and pain. For example, comparing heart rate variability and salivary or urine cortisol levels (35, 38, 39) between the two groups or using quantitative sensory testing (QST) (such as mechanical threshold and thermal threshold evaluation) (11) could decrease the subjectivity of pain and anxiety assessments. Another potential method of pain assessment would be the application of a Grimace Scale that was recently developed in cats to assess acute pain. This scale tends to be unaffected by anxiety behaviors and could facilitate the assessment of dogs without requiring any manipulation (40). Unfortunately, this method of pain assessment is yet to be developed in dogs.

One last limitation is that multiple methods of EE (social enrichment, physical enrichment, and auditory and olfactory stimulations) were evaluated at the same time, which prevents the identification of one single method that would be most effective in reducing post-operative pain and anxiety in dogs. One study suggests that not all commonly used EE methods are equally effective (41). Therefore, it is possible that the observed beneficial effects were the result of only one or a combination of EE measures, but not all of them. Additionally, some of the methods used might have canceled out each other's effects. For example, the use of odor stimulation with essential oil might have interacted with the effect of DAP by detracting from the smell and potentially reducing its efficacy. Additional studies are needed to investigate the effect of each individual method post-operatively and identify the method that is more likely to have a positive effect when implemented in a perioperative context. It would be interesting to look at other behaviors that can reflect an animal's wellbeing, in particular, the amount of sleep that these animals get during hospitalization.

In conclusion, the use of perioperative environmental enrichment methods as part of a multimodal analgesic protocol warrants further studies to determine whether they could be beneficial in improving pain and anxiety following surgery. Management of anxiety with pharmaceutical and non-pharmaceutical therapies can improve the wellbeing of patients after surgery, as they appear to be beneficial for the return of appetite. Finally, more objective methods to assess pain and anxiety are warranted in the field of veterinary medicine in the future.

## Data availability statement

The raw data supporting the conclusions of this article will be made available by the authors, without undue reservation.

## Ethics statement

The animal study was reviewed and approved by Institutional Animal Care and Use Committee of the University of Tennessee (Protocol No. 2604_0418). Written informed consent was obtained from the owners for the participation of their animals in this study.

## Author contributions

EP performed all the assessments, collected and organized all the data, drafted the material and method part of the manuscript. JA participated in the study design and the choice of the different methods of environmental enrichment, revised, and approved the final version of the manuscript. CS assisted with data analysis and statistics. AC designed the study, analyzed the data, wrote, revised, and approved the final version of the manuscript. All authors contributed to the article and approved the submitted version.
