# Supplementary material for: Evaluation of different methods of environmental enrichment to control anxiety in dogs undergoing hemilaminectomy after acute intervertebral disc extrusion: a randomized double-blinded study
Source: Front Vet Sci. 2023 May 30;10:1124982. doi: 10.3389/fvets.2023.1124982 (PMC10267703; doi:10.3389/fvets.2023.1124982)
Supplement: Supplementary file 1 [file Data_Sheet_1.PDF]

SHORT FORM OF THE GLASGOW COMPOSITE PAIN SCALE

Dog's name ________________________________

Hospital Number __________ **Date** / / **Time**

Surgery Yes/No (delete as appropriate)

Procedure or Condition____

____

In the sections below please circle the appropriate score in each list and sum these to give the total score.

A. Look at dog in Kennel

Is the dog?

(i)

Quiet	0
Crying or whimpering	1
Groaning	2
Screaming	3

(ii)

Ignoring any wound or painful area	0
Looking at wound or painful area	1
Licking wound or painful area	2
Rubbing wound or painful area	3
Chewing wound or painful area	4

In the case of spinal, pelvic or multiple limb fractures, or where assistance is required to aid locomotion do not carry out section **B** and proceed to **C**
Please tick if this is the case ☐ then proceed to C.

B. Put lead on dog and lead out of the kennel.

When the dog rises/walks is it?

(iii)

Normal	0
Lame	1
Slow or reluctant	2
Stiff	3
It refuses to move	4

C. If it has a wound or painful area including abdomen, apply gentle pressure 2 inches round the site.

Does it?

(iv)

Do nothing	0
Look round	1
Flinch	2
Growl or guard area	3
Snap	4
Cry	5

D. Overall

Is the dog?

(v)

Happy and content or happy and bouncy	0
Quiet	1
Indifferent or non-responsive to surroundings	2
Nervous or anxious or fearful	3
Depressed or non-responsive to stimulation	4

Is the dog?

(vi)

Comfortable	0
Unsettled	1
Restless	2
Hunched or tense	3
Rigid	4

© University of Glasgow

Total Score (i+ii+iii+iv+v+vi) = ______

Date: **If Total Score is greater than 5 (4 when not carrying out section B) give analgesics**

Time:	1	2	3	4	5	6	7	8	9	10	11	12	13	14	15	16	17	18	19	20	21	22	23	24
A1																								
A2																								
B1																								
C1																								
D1																								
D2																								
Total																								

Notes:

Date: **If Total Score is greater than 5 (4 when not carrying out section B) give analgesics**

Time:	1	2	3	4	5	6	7	8	9	10	11	12	13	14	15	16	17	18	19	20	21	22	23	24
A1																								
A2																								
B1																								
C1																								
D1																								
D2																								
Total																								

Notes:

Date: **If Total Score is greater than 5 (4 when not carrying out section B) give analgesics**

Time:	1	2	3	4	5	6	7	8	9	10	11	12	13	14	15	16	17	18	19	20	21	22	23	24
A1																								
A2																								
B1																								
C1																								
D1																								
D2																								
Total																								

Notes:

Date: **If Total Score is greater than 5 (4 when not carrying out section B) give analgesics**

Time:	1	2	3	4	5	6	7	8	9	10	11	12	13	14	15	16	17	18	19	20	21	22	23	24
A1																								
A2																								
B1																								
C1																								
D1																								
D2																								
Total																								

Notes:

Downloaded from www.isvra.org (Ref. Reid J et al. Animal Welfare 2007; 16(S):97-104)
